# It Takes a Village: Using Network Science to Identify the Effect of Individual Differences in Bilingual Experience for Theory of Mind

**DOI:** 10.3390/brainsci12040487

**Published:** 2022-04-09

**Authors:** Ester Navarro, Vincent DeLuca, Eleonora Rossi

**Affiliations:** 1 Center for Applied Brain and Cognitive Sciences, Tufts University, Medford, MA 02155, USA; 2 Department of Language and Culture, The Artic University of Norway, Hansine Hansens Veg 18, 9019 Tromsø, Norway; 3 Department of Linguistics, University of Florida, Gainesville, FL 32611, USA; eleonora.rossi@ufl.edu

**Keywords:** theory of mind, bilingualism, social networks

## Abstract

An increasing amount of research has examined the effects of bilingualism on performance in theory of mind (ToM) tasks. Bilinguals outperform monolinguals in ToM when comparing groups. However, it is unclear what aspects of the bilingual experience contribute to this effect in a dynamic construct like ToM. To date, bilingualism has been conceptualized as a dichotic skill that is distinct from monolingualism, obscuring nuances in the degree that different bilingual experience affects cognition. The current study used a combination of network science, cognitive, and linguistic behavioral measurements to explore the factors that influence perspective-taking ToM based on participants’ current and previous experience with language, as well as their family networks’ experience with language. The results suggest that some aspects of the bilingual experience predict task performance, but not others, and these predictors align with the two-system theory of ToM. Overall, the findings provide evidence for the extent to which individual differences in bilingualism are related to different cognitive outcomes.

## 1. Introduction

Bilingualism has been extensively studied in the linguistic and cognitive literature, especially in relation to performance effects in tasks of cognitive control, inhibition, reading comprehension, or perspective-taking, among others. However, most research to date has conceptualized bilingualism from an all-or-none perspective, that is, dividing participants into bilinguals or monolinguals with little room for variability [1]. More recently, neurobehavioral models of bilingual language use have begun to consider bilingualism as a dynamic trait that varies along a continuum of how bilinguals utilize their languages in a more fine-grained fashion [2], including variation in factors like sociolinguistic diversity background and culture [3,4,5,6,7]. In other words, researchers are increasingly recognizing the role of individual differences in bilingualism and are taking that variability into consideration.

In the past decades, efforts have been made to create extensive language and language use experience questionnaires to capture self-reported individual measures of variability in bi/multilingual (or even monolingual) experience (e.g., [8,9]). These tools have proven foundational to collect more detailed information on how individuals use their languages in different moments of life and social contexts. However, they do not (or very scarcely) collect information on language use beyond the individual self, neglecting to collect information on communication behaviors between the individuals and their interlocutors, and/or the linguistic and communicative behaviors of interlocutors who interact with the speaker. To fill this gap, researchers in the field have begun to incorporate network science as a way to examine the interactions among individual relationships and cognitive or linguistic behaviors. For example, researchers are using personal networks (a subfield within network science) to understand the degree to which bilinguals use their languages in different contexts or with different interlocutors [4,10] beyond variability in use at the single individual level.

This new perspective on bilingualism has the potential to shed light on research findings that present conflicting results. One example are studies that examine the effect of bilingualism on theory of mind. Past research has highlighted that bilinguals outperform monolinguals in tasks of theory of mind largely due to differences in metalinguistic ability [11,12]. Specifically, bilinguals tend to present advantages in metalinguistic performance from a young age due to their experience interpreting and manipulating different languages in different social situations. However, it has not been studied whether bilinguals’ theory of mind performance can be predicted by other sociolinguistic factors proper of bilingualism using social science network techniques. By taking this approach, we could identify the components of language experience that can affect one’s ability to understand others’ perspectives, showing that understudied language factors are likely to contribute to cognitive performance. In this novel study, we used social network science and behavioral methods to study linguistic, sociocultural, and cognitive responses from 89 young adults with varying degrees of bilingual experiences. The goal was to explore the predictors of perspective-taking theory of mind and metalinguistic awareness, above and beyond a monolithic view of bilingualism.

### 1.1. New Approaches to Bilingualism

Bilingualism has been traditionally conceptualized as a dichotic phenomenon contrary to monolingualism. More concretely, the general convention in fields like neurolinguistics, psycholinguistics, and cognitive science has been to compare bilinguals to monolinguals as two sides of the same coin, considering monolingualism the default state, and bilingualism an extension of this experience [13]. This view largely differs from many areas of psychology, including personality, intelligence, and emotion regulation, that study individual differences in performance by examining variance between and within participants. Instead, until recently, the field of bilingualism has largely focused on how speaking more than one language affects various neural and cognitive outcomes, discarding individual variability in language experience as noise. However, in recent years, researchers have begun to describe bilingualism as a more fine-grained construct, increasingly focusing on the degree of variability across language experiences and how that variability influences other linguistic, social, and cognitive abilities [3,4,7,10]. A key example of this effort is represented by the Adaptive Control Hypothesis (ACH) [2]. ACH’s central idea is that bilinguals accommodate neurocognitive engagement depending on the dynamics of different conversational contexts. Even though real bi/multilingual language use can be more complex, ACH proposes that bilinguals might interact in a single language alone (i.e., the two languages are kept separate), a dual language context (i.e., the speaker might switch between languages in the course of a conversation, but not necessarily switch within a sentence), or a dense code-switching environment (i.e., when bilinguals switch languages fluidly within the same sentence). Critically, depending on the speaker’s varying interactional context, the bilingual’s neurocognitive demands will engage adaptively. For example, in a dual language context, ACH predicts the engagement of neural circuits important for conflict monitoring and interference suppression. In a dense code-switching environment, instead, there will be adaptation involving neural regions necessary to mediate the late retrieval and activation of both languages at the same time. This adaptive model has been validated by other recent studies that have demonstrated how differential interactional contexts and environments shape bilingual executive functions’ recruitment [12,13]. Taking this broader variability perspective, an increasing number of studies have now examined the effects of individual differences in bilingual experience on a number of cognitive abilities, including different aspects of executive functions, such as reading comprehension, and mentalizing [10,14,15,16,17,18,19,20,21,22,23]. 

Even though recent research has started to address differences in linguistic contexts and their differential demands on neurocognitive adaptation [12,13], one especially relevant source of variability that has been largely overlooked in bilingualism research is the social context and varying functional demands of language use (e.g., see [6]), which is often studied in areas like sociolinguistics and linguistic anthropology. For example, young Spanish–English bilinguals living in a predominantly Puerto Rican neighborhood in New York City not only used Spanish and English differentially with different people (e.g., Spanish with parents, English with siblings), but the content and context of the conversation also constrained their language choice (e.g., they sometimes used English with parents to discuss work and school). This was described in the Complementary Principle [24,25,26,27,28], which states that bilinguals use their languages for various purposes, across various domains of life, and with various people [29,30,31,32]. In fact, a growing body of research has demonstrated that bilingualism as a trait can vary consistently, including the age of acquisition, proficiency, number of languages, frequency of language use, tone, context, formal training, cultural immersion, personal motivation, and cultural appreciation, among many others [24]. One underexplored aspect of bilingualism that has been the subject of several studies in recent years is the influence of one’s sociolinguistic network (Complementary Principle [14]) on neurocognitive outcomes. More concretely, the question has been asked of how individual differences in the sociolinguistic background of a speaker can influence performance in behavioral and linguistic outcomes, regardless of whether they consider themselves to be bilingual. In our ever-growing multilingual and multicultural societies, this is a query that could help explain the effects that having a dynamic language experience provides to an individual, but also the extent to which being bilingual can vary between and within people. As mentioned above, in recent years, a small but growing body of work has begun to examine the role of social context in bilingual outcomes from both a theoretical [2] and methodological perspective [5,12,13,33,34]. The predictions and results from this work all suggest that social context is at least deterministic of language use patterns [34], if not explanatory in its own right of neurocognitive outcomes associated with bilingual experience [5,33]. 

Individual-level factors are, therefore, deterministic for bilingual language exposure and for language engagement over time, thus becoming critical factors for understanding bilingual outcomes, including linguistic, behavioral, and neurocognitive effects [2,34,35,36]. In other words, as opposed to an actual state, bilingualism should be understood as a spectrum; where an individual’s experiences place them along this spectrum determines what outcomes in language and mind/brain can be expected. Broader level variables such as the social context and linguistic environment may also play a modulatory role in shaping these adaptations. 

### 1.2. Theory of Mind and Bilingualism

Theory of mind (ToM) is the ability to understand the beliefs, knowledge, and intentions of others based on their behavior. The term was first coined by Premack and Woodruff [35] to refer to chimpanzees’ ability to infer human goals, and it was quickly adopted by psychologists to study humans’ ability to infer and predict the behavior of others. Numerous theoretical approaches have been proposed to explain the processes underlying ToM. Generally, theories of ToM can be largely classified according to two features, namely, (a) whether they describe ToM in terms of domain-general vs. domain-specific processes, and (b) whether they view ToM as a subset of specialized modules. Most theories fall within the so-called Competence–Performance framework [36,37,38,39,40,41,42], which debates whether ToM is developed as a result of the trial-and-error interactions with one’s environment or whether it is dependent on the development of inhibitory and cognitive control processes during early childhood. However, while the Competence–Performance frameworks attempt to explain ToM from a developmental perspective, more recent theories focus on describing ToM from a neurobiological perspective. For this reason, more recent accounts are rooted in neuroimaging research and describe the possible neural areas that contribute to ToM.

One popular account that considers ToM beyond the Competence–Performance debate is the two-system theory proposed by Apperly and Butterfill [43]. The two-system theory is largely derived from behavioral and neuroimaging research that distinguishes between implicit ToM (i.e., fast and automatic) and explicit ToM (i.e., slower and deliberative ToM) [43,44,45]. The two-system theory suggests that ToM ability can be better explained by a dual cognitive mechanism that can account for both the executive function–ToM relationship that has been well documented in the literature [46,47] and conceptual changes based on experience and/or context. In other words, this theory provides a more comprehensive and parsimonious description of ToM ability. The dual-system view is based on the classical dual-process theory of decision making that proposes that human cognition is defined by a distinction between effortless, intuitive, automatic processes (System 1) and effortful, deliberative, operational processes (System 2) [48,49,50,51] System 1 processes are thought to be autonomous and automatic because they have been practiced extensively and give rise to heuristics and biases; most times, heuristics and biases allow individuals to reach a correct conclusion in an effortless manner, but sometimes they lead to erroneous or irrational responses, such as substituting hard-to-remember attributes for easier, but less accurate, ones [52]. Type 2 processes are non-autonomous (and therefore cognitively slow) and “computationally expensive” ([53], p. 440). For this reason, they are not engaged as often as Type 1 processes, but can be used to override Type 1 responses when heuristics fail. Failures to override inadequate Type 1 responses result in the incorrect use of heuristics and biases. According to Apperly and Butterfill, in ToM, System 1 is used by infants and young children before they have full control over their ToM, but also by adults when the situation does not require effortful processing, such as when there is no perspective conflict or when the perspective conflict has been encountered in the past so that it becomes an automatic response. Following this account, ToM would present itself as a largely effortless process, except when taxing conflicting perspectives arise. System 1 thus precedes and contributes to System 2, a fully formed ability used to comprehend other mental states that requires effortful processing. While the researchers do not directly address the possibility of there being individual differences in the use of these two systems, the two-system view naturally allows for these differences to exist, as some individuals might be able to override inappropriate System 1 responses more efficiently by engaging other cognitive or noncognitive (e.g., experience-based) processes.

Yet, despite alternative theoretical accounts proposed to explain ToM performance, the Competence–Performance debate is still one of the most common frameworks. This debate cannot be easily resolved by studying standard samples because both frameworks make the same predictions. That is, theories from both frameworks propose that ToM develops between the ages of 3 and 5 due to different underlying processes (i.e., experience and cognitive control, respectively). However, Wellman [54] suggested that studying individual differences in ToM could help researchers understand the processes underlying ToM performance. Individual differences, such as engaging in social pretend play, having siblings, or growing up bilingual have been found to affect the development of ToM. Specifically, bilinguals have been shown to outperform monolinguals in ToM [55,56,57,58], and this effect seems to be stable across tasks and not subject to publication bias (see [59] for a meta-analysis). 

Understanding the processes underlying bilinguals’ ToM performance could help explain the processes that are engaged in ToM ability. Specifically, bilinguals present differences in the factors that influence various cognitive and linguistic mechanisms, such as metalinguistic awareness and vocabulary size, that are predictors of ToM performance [11,60]. Bilinguals also show differences in a number of different neural efficiency measures. For example, older adults who are bilinguals present stronger cognitive and linguistic efficiency [61]. Differences in bilinguals’ responses to cognitive ability tasks might reflect variation in the type of cognitive processes used by bilinguals. This suggests that individual differences in ToM also may be related to variation in bilinguals’ processing when responding to ToM tasks.

Bialystok and Senman [56] suggested that the reason why bilinguals outperform monolinguals in ToM is that bilinguals possess domain-general executive function advantages over monolinguals on tasks that involve ambiguous and conflicting information thanks to their experience controlling both of their languages [62,63,64,65,66], thus supporting the Performance framework. However, Goetz [58] proposed, instead, that bilinguals’ conscious switching between languages could be the result of bilinguals’ awareness of the languages that people around them can and cannot speak [67]. This, in turn, could translate into improved metalinguistic awareness, that is, awareness that objects and events can be represented in more than one way [61,68,69], helping bilinguals comprehend that people have different mental states at an earlier age. Studies have found that young bilingual children are able to switch to the appropriate language of their interlocutor [70,71], suggesting that bilinguals may be more aware of the fact that other people have different mental states than are monolingual children [67]. Thus, Goetz proposes that advantages in metalinguistic awareness increase ToM performance, supporting the *Competence* framework.

Evidence for both of these hypotheses has been put forward. For example, Goetz [58] examined the ToM performance of bilingual and monolingual 3–4-year-olds in two temporally separate sessions and found that bilinguals performed better than monolinguals in most of the tasks in the first session, but the difference disappeared in the second session. Goetz proposed that this was due to the amount of practice the children received in the first session, thus suggesting that bilinguals have more ToM “practice” as a result of their interactions with people who speak different languages, but this difference can be overcome if monolinguals practice their ToM. On the other hand, Kovacs [55] administered 3-year-old monolingual and bilingual children a modified language-based task (i.e., requiring metalinguistic abilities) and a standard false-belief task (i.e., not requiring additional metalinguistic abilities). Kovacs hypothesized that bilinguals should have performed better in the modified language-based task than in the standard task because the modified task depicted a language-switch context that should have facilitated ToM if bilinguals indeed have improved ToM skills due to metalinguistic awareness. However, Kovacs found that bilingual children outperformed monolinguals on both tasks, not just in the modified task. According to Kovacs, this indicated that bilinguals’ performance is not due to a metalinguistic advantage, but instead could be due to a domain-general advantage over monolinguals.

However, more recent studies have considered that bilinguals might use differential processes when completing ToM tasks, rejecting the dichotomy of bilinguals having an advantage in either executive function or metalinguistic awareness. For example, Diaz and Farrar [11] found that executive function predicted ToM performance a year later for a group of monolingual (but not bilingual) preschoolers, while metalinguistic awareness predicted ToM performance a year later for bilinguals (but not monolinguals). Similarly, Buac and Kaushanskaya [72] found that executive function predicted ToM performance for monolingual but not bilingual children, while metalinguistic ability predicted ToM performance for bilingual but not monolingual children. These findings suggest that instead of bilinguals having a quantitative advantage (i.e., bilinguals use the same cognitive processes as monolinguals, but they do so more effectively), bilinguals could have a qualitative advantage, that is, bilinguals and monolinguals might be using, to an extent, different mechanisms when completing ToM tasks with one set of mechanisms producing superior results in some contexts. 

If the processes underlying ToM can vary based on specific individual experiences, such as being bilingual, then adults’ performance should reflect these variations. Indeed, adults (monolinguals) show individual differences in different types of ToM performance [73,74,75,76]. In addition, ToM seems to develop throughout the lifespan and becomes increasingly less effortful in young adulthood [77], suggesting that the processes used to engage ToM in childhood gradually change in adulthood. In fact, compared to adults, older children and adolescents show differential engagement in neural networks dedicated to control when responding to ToM tasks (e.g., right temporoparietal junction), suggesting that ToM is not an immutable ability (e.g., [77]).

While there is evidence that adult bilinguals outperform monolinguals on ToM tasks [76,78,79,80], just like for studies with children, these effects have been found after dividing participants into monolithic groups of either bilinguals or monolinguals. However, as described in the section above, this largely artificial divide is likely masking nuances in the degree to which bilingualism-dependent factors predict ToM performance. For example, no study to date has examined whether individual differences in bilingualism predict ToM differently, nor whether different subcomponents of the bilingual experience vary in their prediction ability of ToM. If this is the case, then individual differences in bilingualism could shed some light on the complexity of the processes involved in ToM, and which processes bilinguals preferentially engage.

As introduced above, network science is a methodological tool that is gaining traction among researchers in bilingualism and neuro/psycholinguistics. Network science is the study of the pattern of relationships, behaviors, or experiences among social actors. Personal or social network research falls under this larger field of network science. In a social network study, an individual’s network refers to a set of actors (or nodes) (e.g., school friends, coworkers) and the ties among them (or edges) (e.g., immigration status, education) [81]. The advantage of social networks is that the relationships among a person (i.e., ego) and their individual personal connections (i.e., alters), as well as among the persons’ network (alter–alter ties), can be examined and compared. For example, Tiv et al. [10] compared the network profiles of a group of bilinguals in two different areas and found that language betweenness was a predictor of performance in a mentalizing task in highly linguistically diverse regions, but not less linguistically diverse regions. Social network research is largely used in areas of sociology and linguistics, although other types of social network, such as psychometric network modeling, are also increasingly being used in the field of clinical and cognitive psychology, and psychometrics. Social networks have been studied to understand such various topics as collaborative relationships in the workplace [82], migration flows between countries [83], and, more recently, to explore communication patterns among bilingual speakers in Canada [7].

### 1.3. Current Study

Given the above discussion, the goal of this study is to understand whether and to what extent differences in bilingual experiences for the individual (ego), as well as their broader networks’ linguistic behavior (alters, and alter–alter ties) differentially predict ToM performance and, therefore, provide a wider understanding of the importance of measuring components of the sociolinguistic experience to predict cognitive outcomes. Specifically, we aimed to examine (a) whether ego-related variables and alter-related variables of bilingual experience differentially predict ToM as assessed by the director task, and (b) whether metalinguistic awareness predicts ToM performance once other aspects of the bilingual experience are measured.

The current study uses a combination of social network science, cognitive, and linguistic predictors to explore the factors that influence theory of mind (ToM) performance based on participants’ current and childhood experience with language, as well as their networks’ experience with language. 

In addition, the design of this study enables us to understand whether variability in linguistic behavior and social/linguistic context reported from childhood, for both the individual and their network, has repercussions on synchronic performance in ToM, thus opening a window to understand the connection between language use in childhood and its potential long-lasting effects on cognitive performance later in life. Scripts and data are available at: https://osf.io/azmpk/ (accessed on 6 April 2022).

## 2. Materials and Methods

### 2.1. Design and Participants

An online sample of participants was recruited via email diffusion through colleagues and researchers working across areas of the United States with high linguistic diversity. Approximately 100 participants completed all tasks. After removing incomplete or duplicated responses and participants who did not follow the instructions, the final sample size was *N* = 89. The participants were part of undergraduate courses at various higher education institutions in Southern California, Texas, and Florida, who completed the study online in exchange for course credit. All participants were invited to participate regardless of bilingual identification or any other linguistic criteria to increase diversity. None reported being color blind and all participants reported having normal or corrected-to-normal vision. All details about the sample are summarized in Table 1. All participants completed a perspective-taking ToM task, a metalinguistic awareness task, and a social network questionnaire. 

### 2.2. Measures

#### 2.2.1. Director Task 

The director task is a perspective-taking-based task of theory of mind often used to assess adults’ performance, as it assesses both the mentalizing and non-emerging aspects of ToM [84]. The director task taps perspective-taking components of ToM, rather than perceptual or emotional dimensions of ToM. This perspective-taking dimension has also been largely studied in adult non-clinical populations as it is related to cognitive inhibitory processes [85,86,87,88]. 

A typical director task includes two conditions (Director, No Director) and two trial types (Experimental, Control). The stimuli are set up in a 4 × 4 shelf containing eight different objects arranged in different positions. In the Director condition, an avatar called the director is placed behind the shelf. Some of the compartments in the shelf are occluded from the director’s view so that only the participant can see those objects. The director stands behind the shelf, so that only the objects in the open compartments are visible to him. The participant is then asked to follow the director’s instructions, such as “the yellow sock” or “the small cup” and click on the correct object within the shelf. In the *Director* condition, participants were shown the shelf from the perspective of the director and were explicitly told that the director cannot see objects in the occluded compartments. This condition assesses theory of mind because the participant has to remember that the perspective of the director is not the same as theirs. In the *No Director* condition, participants are shown the same shelf, but the director is not behind it. Instead, the participants are given a strategy: the participants are told to ignore all objects placed in the slots with red backgrounds. Both conditions include two types of trials: (i) experimental trials, where the participant must select the correct response (i.e., the target), while ignoring an egocentric competing object that only the participant can see from their perspective, and (ii) control trials, where there is no egocentric competing object. A total of 16 control and 16 experimental trials appear in a pseudorandom intermixed order throughout the task where filler trials are also presented, and the order of the presentation of the stimuli is counterbalanced across participants. The participants also complete a practice trial before the task.

In this study, the director task was implemented in a mouse-tracking paradigm using the online experiment builder FindingFive (https://www.findingfive.com/) (accessed on 6 April 2022). The director task often elicits small effects in accuracy and no effect in reaction time (e.g., [77,80]) largely due to the slow nature of the manual responses. For that reason, in this study, we set out to investigate the degree of error in manual deviation from the correct target that a participant demonstrated or (signed) maximum absolute deviation (MAD) [89]. MAD is an indicator of curvature in mouse-tracking analysis used to assess the degree of deviation from a target. Positive MAD indicates the deviation is largest above the direct path (in the direction of the non-chosen alternative) and negative MAD indicates that the point of strongest deviation occurs below the direct path. The curvature of the response trajectory is used to assess the degree of its attraction towards the non-chosen option and it is assumed to be an indicator of the difference in activation between the non-chosen and the chosen option [90]. This metric reflects the extent to which the selection of an item provokes response conflict. In this study, MAD was used to assess the degree to which the participants were first attracted towards the decoy target, even if they did not end up selecting it, following the original paradigm by Keysar, Lin, and Barr [74].

#### 2.2.2. Metalinguistic Awareness

Metalinguistic awareness was measured using the tasks developed by Cartwright et al. [91] for adult samples. The tasks assess the contributions of metalinguistic awareness and cognitive flexibility. The two measures of metalinguistic awareness correspond to non-semantic aspects of cognitive flexibility and are thought to assess the relative contributions of particular aspects of metalinguistic awareness and cognitive flexibility to differences between good and poor comprehenders. The first subtest of the task is the graphophonemic awareness test, which consists of 30 phoneme counting problems in which participants have to count the phonemes in printed words (e.g., *filth* contains four phonemes). The second subtest was the syntactic awareness test, which consists of a 10-item word order correction task in which participants must reorder sets of words into syntactically correct sentences. Multiple solutions are possible for each set of words (e.g., “the words dog is small the timid” could be reordered as “The timid dog is small” and “The small dog is timid”). The scores are the total number of appropriate sentences generated across the 10 sets of words.

#### 2.2.3. Social Network Questionnaire

In this study, a novel social network survey was constructed following rigorous social network analysis methodology [81] to examine the language experience properties of the participants and their networks. For this purpose, an extensive questionnaire was built using validated tools available in the literature (i.e., [8,92,93]), as well as novel questions, to gather as much information as possible about the language experience, switching tendencies, context, use, and childhood use of the participant or *ego* (known as ego-level data). In addition, questionnaires regarding language use and experience with members of the ego’s immediate family in the present and during childhood (known as alter-level data) as well as the language use and experiences that the participant’s alters had among each other in the present and during the participant’s childhood (known as alter ties-level data) were created. 

Each participant first responded to a few short demographic questions, including age, gender, and education. Then, the participants began the first section of the survey (adapted from Marian, Blumenfeld and Kushanskaya [92], and Anderson et al. [8]), where they named first, second, and other (if applicable) languages they know based on dominance, the age(s) at which they began speaking those languages, their cultural identification, and the number of years they have lived in a country where the languages are spoken. Next, they provided percent estimates (on a scale from 0 to 100) of the average use for each reported language among different groups (family, friends, etc.) and for different activities (shopping, restaurants). They then rated their fluency in each language (on a scale from 0–10). Next, they provided information about the languages they used in different life stages, including infancy, high school, and college. Finally, they reported their use of language switching and tendencies across different groups and contexts (adapted from Rodriguez-Fornells et al., 2012 [93]). After the participants completed the ego section of the survey, they began the questions regarding the alters and their relationships. The participants were asked to provide the names of up to five members of their family with whom they interact often and were then asked questions about each of them, as well as about each of the relationships among them. For example, when a participant names “Mom” as one of their alters, they are then asked to provide the languages they speak with “Mom”, the languages they spoke with “Mom” as a child, and whether they switch languages when speaking with “Mom”. After the participant answered all questions about each of their alters, they were asked questions about the alter ties. For example, a participant will be asked to indicate whether “Mom” and “Dad” talk to each other in the present and during the participant’s childhood, what language(s) they use, and whether they switch languages now and in the past. The participants answered these questions for each alter pair (e.g., “Mom and Dad”, “Mom and Sister”, “Dad and Sister”). The questions were then averaged within each theme (e.g., language use, switching, total languages, alters languages, alters switching) to create composite scores that summarized each set of questions. The survey presented adequate internal consistency (α = 0.75).

By collecting the responses from these three levels of bilingual experience (ego, alters, and alter ties), we expected to create a multidimensional set of variables that comprise the bilingual experience of an individual above and beyond information regarding their individual current use. This degree of information provides a deeper understanding of the dynamics of bilingual experience from an individual differences perspective that does not dichotomize participants based on any given variable. Overall, positive responses to the social network survey indicated more use of the language or more interaction with a given alter, whereas less use or less interaction resulted in lower scores. The survey included over 100 questions about the ego, alters, and alter ties.

### 2.3. Procedure

Students were invited to participate in the study via recruiting emails sent to colleagues and professors in the United States. The participants were offered course credit in exchange for participation. The study was conducted fully online and asynchronously as it was shared across multiple states in the United States in an attempt to obtain as much variability in the responses as possible in terms of bilingual experience. The students were largely based in higher education institutions in Southern California, Texas, and Florida. The students were first directed to a landing page created by the researchers to explain the purpose of the study, as well as to provide instructions on how to complete the study. Once the participants entered the site, they were asked to complete an online consent form. The participants then proceeded to complete the director task, the metalinguistic task, and the network questionnaire via links available on the website. At the end of the study, the participants were directed to a separate survey where they entered their personal information to obtain credit. All responses were anonymized and de-identified. All participants completed the tasks in the same order, as it is the norm in studies of individual differences to avoid biasing effects within the tasks, such as fatigue.

### 2.4. Analyses 

The participants responded to approximately 112 questions of the social network questionnaire, the metalinguistic task battery, and the director task. First, composite scores averaged across questions were created based on the information asked in those questions so that they would be collapsed into individual constructs. That is, for each set of questions that participants answered for a given topic (switching with friends, coworkers, etc.), a composite score was created (e.g., ego switching). The composite predictors extracted from the questionnaire were: the ego’s L1 and L2 average use, the cultures reported by the ego, the ego’s reported L1 and L2 fluency, the languages spoken between egos and alters currently and during childhood, the languages spoken among alters currently and during childhood, the number of language spoken throughout the ego’s lifespan, the ego’s language switching tendencies among different groups, and the alters’ language switching tendencies currently and during childhood with the ego and with other alters (see Table 2). In addition to the social network data, a metalinguistic composite score was calculated based on participants’ correct responses to the metalinguistic sub-tasks. Finally, as discussed above, we calculated the degree of error in manual deviation from the correct target that a participant demonstrated or (signed) maximum absolute deviation (MAD) [89], an indicator of curvature in mouse-tracking analysis used to assess the degree of deviation from a target. After inspection, the MAD score presented a negatively skewed distribution, therefore it was log-transformed in order to meet normal distribution assumptions. Based on the correlational analyses and the theoretical accounts of ToM, subsets of constructs were selected as predictors of ToM performance. Specifically, given previous studies that tested the Competence and Performance frameworks of ToM, it was expected that the switching tendencies and average use of two languages (performance) and childhood language use among alters and languages spoken throughout early life (competence) would be relevant candidates for predictors of ToM. For these reasons, we selected a subset of theoretically relevant predictors to include in the model. To ensure that the selection of variables was based on an objective approach, in addition to the theoretical motivation, we conducted a stepwise regression analysis to explore whether the model selected was indeed the most adequate model across all variables. Stepwise regression or selection consists of iteratively adding and removing predictors in the predictive model to find the subset of variables in the data set, resulting in the best performing model. Using this approach, we identified the best model containing Mean L2, Ego Switching, Alter Languages in Childhood, Alter Ties Languages, and Alter Languages in Childhood. This output model largely followed our theoretical approach; however, our model included two other variables that we considered were relevant in theoretical grounds. For this reason, we consider that variable selection was appropriate in this model. In addition, a multicollinearity examination revealed no problematic variables in the model, based on variance inflation factors (VIF) and tolerance analysis (all variables < 4). VIFs measure the inflation in the variances of the parameter estimates due to collinearities that exist among the predictors. Tolerance analysis represents the percent of variance in the predictor that cannot be accounted for by other predictors. A VIF exceeding 4 usually warrants further investigation, and VIFs exceeding 10 are signs of serious multicollinearity. As mentioned, none of our variables presented problematic values. Multiple regressions were conducted to explore the effects of each bilingual experience variable on theory of mind performance.

## 3. Results

The descriptive statistics and correlations are presented in Table 2 and Table 3. A power analysis was not conducted a priori because the current sample was a subset of a larger sample where participants completed fewer tasks. Thus, to ensure that the analysis was sufficiently powered, a post hoc power analysis was conducted using G*Power. The analysis showed that a multiple regression examining the main effects with a sample of 89 and seven predictors (α = 0.05) for a medium-to-small size effect would approximately elicit *β* = 0.86 power, suggesting that the sample size recruited was sufficient for this analysis. 

**Figure 1:**
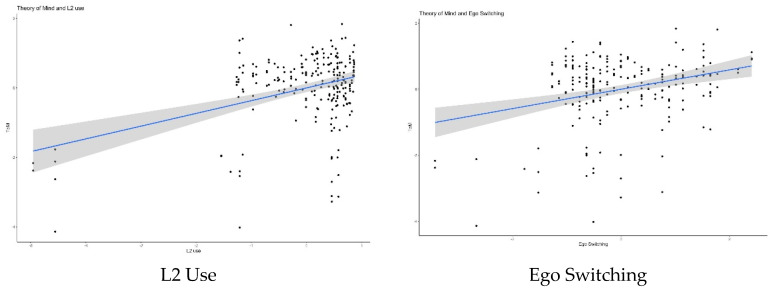

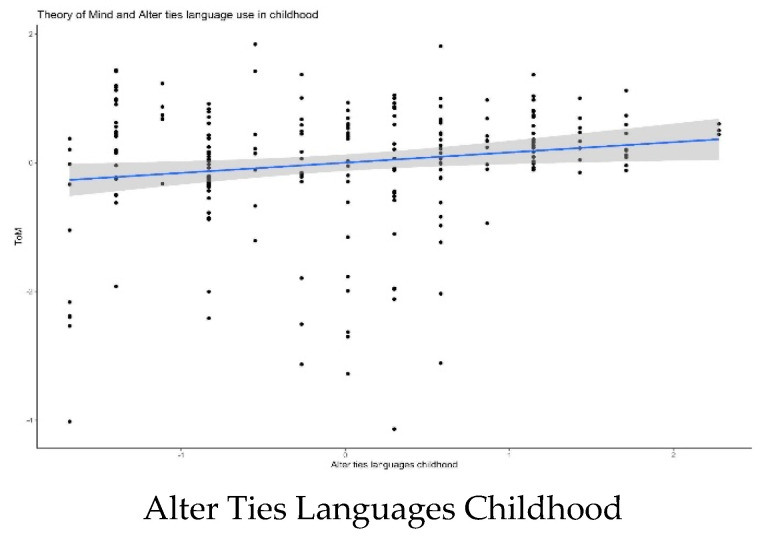
Significant effects of multiple regression. L2 use (**upper left**), ego switching (**upper right**), and alter ties languages in childhood (**bottom**) as predictors of ToM.

A multiple regression was conducted, with ego’s switching tendencies, languages spoken among alters during childhood, alter’s language switching tendencies with other alters, ego’s languages spoken throughout the lifespan, ego’s average use of second language(s), ego’s overall language fluency, and metalinguistic awareness as predictors of MAD performance in the director task for critical trials (i.e., ToM). The regression results revealed that, of the hypothesized variables, the ego’s mean language use and the ego’s switching tendencies were significant predictors of ToM, replicating previous findings in the literature and suggesting that regular use of a second language, and regular switching between languages, are relevant factors in ToM performance. This finding provides support for the Performance framework of ToM. In addition, the languages that the ego’s alters spoke during the ego’s childhood were also a significant predictor of ToM, possibly indicating that experiencing multiple languages, especially among people in one’s immediate family, might also be relevant for ToM performance up to young adulthood. This, in turn, provides support for the Competence framework of ToM (see Table 4). Interestingly, in these results, metalinguistic awareness was not a significant predictor of ToM, perhaps because the variance that metalinguistic awareness accounts for in ToM performance was, in this case, canceled out by the other predictors. This could indicate that the effect of metalinguistic awareness on ToM might be accounted for by other sociolinguistic predictors. We explored this in an additional analysis. The model accounted for about 18% of variation in ToM, indicating that there is still room for improvement in the prediction of ToM performance. Figure 1 shows the effect of the significant predictors of ToM.

In addition to the main analysis above, we conducted an additional multiple regression to explore the extent to which the current set of predictors are related to metalinguistic awareness. As mentioned above, it is possible that metalinguistic awareness performance is influenced by one’s sociolinguistic experience, especially when multiple languages are learned in childhood. For this reason, the same regression described above was conducted again, this time with metalinguistic awareness as the predictor. The results of this second analysis revealed that *average L2 use* and *languages spoken across the ego’s lifespan* were the only significant predictors of metalinguistic awareness (mean L2 use: B = −0.067, SE = 0.022, *β* = −2.94, *p* = 0.004; languages lifespan: B = −0.144, SE = 0.043, *β* = −3.35, *p* < 0.001). These findings could indicate that in healthy young adults, metalinguistic awareness is more influenced by current use of the language and less by exposure to language earlier in life. This interpretation would be in line with the definition of metalinguistic awareness (i.e., the ability to manipulate and transform language efficiently [93]). This finding could also indicate that, in adults, metalinguistic awareness might be a proxy of the participant’s bilingual skills in the present, but might not reflect the extent to which their metalinguistic experience in childhood modulates ToM. This finding should be further explored in future research.

Overall, the current findings present several takeaways for ToM research. First, ToM, as measured by the director task, was predicted by both the ego’s current experience and use of the language, but also by their alters’ language use during the ego’s childhood—that is, how often the ego speaks their second language, how much they switch between languages, and how often members of their family spoke different languages to each other during the ego’s childhood, but not how fluent the ego currently is (or reports being) in another language or how many languages they spoke while growing up. This suggests that an important component of perspective-taking ToM might be the quality of language use and switching (cognitive processes) and the level of early exposure (sociocultural processes) rather than quantity of languages. Second, at least in adults, it seems that metalinguistic awareness might be a proxy for bilingualism experience, thus not supporting the hypothesis that bilinguals’ performance in ToM is fully moderated by metalinguistic awareness ability. These findings are further discussed below.

## 4. Discussion

The goal of the current study was to examine whether individual differences in bilingualism, including current and past experiences between a person and their network, can help explain performance differences in theory of mind. The findings suggest that different aspects of the bilingual experience can influence perspective-taking ToM to varying degrees, and therefore some aspects of bilingualism affect cognitive outcomes differently.

Multiple theoretical accounts have been proposed to explain the processes underlying theory of mind mechanisms. Theories within the Competence framework propose that ToM is a skill largely dependent on experience, and develops as a result of social interactions. Theories within the Performance framework instead propose that ToM is formed as a result of executive function development, such as inhibiting one’s own perspective and updating one’s knowledge of others’ perspectives. However, these theoretical frameworks exclude the possibility of an interaction of processes related to theory of mind. Apperly and Butterfill’s [43] two-system theory proposes that ToM ability can be better explained by a two-system mechanism in which experience-based heuristics with ToM situations are often used as a default option (System 1), but they can be overridden by a cognitively taxing mechanism when heuristics are not the adequate response (System 2). This dual system can explain why mistakes are sometimes made when considering other people’s perspectives and states of mind, as well as why this can occur more often when cognitive demands are already high [75]. The two-system theory also seems to explain the findings in this study. Specifically, aspects of the bilingual experience that carry a strong cognitive component, such as average use of two languages and switching between languages (tasks that require inhibition, switching, working memory, and updating processes, among others) were predictors of ToM performance, replicating previous findings [10,56,57,58]. This suggests that tasks that involve the continuous use and practice of cognitive mechanisms are relevant for successful ToM performance. While this is in line with the Performance framework, we also found that the languages spoken by the participants’ family members during childhood also influence ToM performance. This suggests that being aware of the fact that people around you speak different languages can influence how easily one understands other people’s perspectives. This finding aligns with predictions of the Competence framework. However, given that the set of predictors in this study are at odds with the Performance–Competence frameworks, the findings seem better explained by the two-system theory of ToM. That is, there are multiple aspects of cognitive and sociolinguistic ability that can influence ToM, and they are likely influenced by context, individual differences, and available resources. This is further suggested by the finding that metalinguistic awareness was predicted by different sociolinguistic aspects than ToM, and that metalinguistic awareness was not a predictor of ToM when other language experience variables were included in the model. Bilinguals’ ToM performance has been explained by suggesting that their improved metalinguistic awareness is used as a bypass of effortful cognitive processes. However, the findings of this study suggest that metalinguistic awareness might be a proxy for the sociolinguistic experiences associated with bilingualism, thus indicating that metalinguistic awareness per se is possibly not the reason why bilinguals outperform monolinguals in ToM, but rather that sociolinguistic factors typical of bilinguals are related to some aspects of ToM use. Overall, the findings of the study show that exploring the aspects of bilingualism that influence performance can help explain what processes are related to ToM, when each of these processes are engaged and how individual differences affect performance.

In addition to the contribution of these findings to ToM accounts, the current results add to a growing body of literature indicating that there is a specificity of neurocognitive adaptations to different language experiences or subcomponents of bilingualism, which have a knock-on effect to domain-general cognitive outcomes. Moreover, the extent of adaptation (measured here via the performance on the director task) is calibrated to the degree of (bilingual) experience. Specifically, the correlations between ToM task performance and L2 use and switching are indicative of neurocognitive adaptations to increased intensity of engagement with bilingual experience, as proposed within neurocognitive adaptive models of bilingualism such as ACH [2], and more recently the UBET framework [94]. The UBET framework posits that two subcomponents of bilingual experience (intensity and diversity of language use and language switching) contribute to greater requirements of executive control systems/processes to handle the associated cognitive load with these experiences. While the UBET framework does not discuss the potential link between language experience and ToM performance specifically, the current results can be interpreted as a general adaptation to increased executive control demands as a function of the intensity of bilingual engagement. As discussed above, the adaptations to increased cognitive demands may thus have a knock-on effect on the ability to recruit the cognitive resources to handle these. As such, it is possible that the correlations to mean L2 use reflect the ability to deploy cognitive resources more readily when required, such as those situations discussed within the two-stage model above [43]. 

Further, the prediction of task performance by alter ties in childhood supports an account of indirect social-level influence on domain-general outcomes within the ego’s language environment, as proposed within the Systems Framework of Bilingualism [34]. Within the framework, the increased bilingual language use present within the ego’s sociolinguistic environment in childhood could increase the need for attentiveness to sociolinguistic situations, thus having a knock-on effect on ToM (also hypothesized by Kloo and Perner [67]). However, as relatively little evidence exists for such connections, further research is required to support this interpretation.

This result also ties in with previous research indicating that indirect (bilingual) language exposure and language environment have effects on several cognitive processes [4,10]. Specifically, as mentioned above, recent work by Tiv and colleagues [10] shows that being exposed to diverse sociolinguistic contexts influences mentalizing processes even when there is no direct bilingual exposure. The results of the present study also indicate a sensitivity to language context (alter ties) and an effect on non-linguistic social cognitive processes derived from these language contexts. Finally, the effects of alter ties in childhood on ToM performance also speak to the need to better capture and quantify broader measures of bilingual experience beyond individual-level engagement, particularly in terms of the details of one’s sociolinguistic environments across the lifespan (e.g., [34]). Here, we submit that measures that are able to capture such levels of experience will be key to future research on bilingualism, including language dynamics across social networks.

This study is not without limitations. While the results of the study are promising, the current social network survey tool represents only a limited version of the network surveys often used in the research literature. Since this study is the first to build a comprehensive social network tool for bilingualism, the questions were limited to family members and included only some aspects of bilingualism, such as the languages spoken with family members and switching tendencies. Future research should expand this work by examining how bilingual communication with other members of their network contributes to individual differences in bilingualism. In addition, because the participants were asked to provide information about language behaviors during their childhood, it is possible that some degree of error was introduced by requiring the recollection of retroactive memories, therefore participants’ responses about their childhood should be considered with caution. However, given that most of the current language and background questionnaire tools include the reporting of behaviors at different points throughout the lifetime, the responses in the current social network are not expected to be more skewed than the overall pool of responses available in the literature. While the results are largely generalized to ToM as a construct, the director task used in this study likely assesses a visual perspective-taking component of ToM, and not other more emotionally-based aspects of the construct. Recently, research has questioned whether the director task measures a specific dimension of ToM or some other cognitive processes, such as mental rotation or selective attention, or possibly a combination of these processes [95]. Future research should examine whether the current findings are replicated in different measures of ToM. It should also be noted that while the sample was determined to be adequately powered for the analyses employed herein, more complex models and analytical approaches will require greater sample sizes. Future research would ideally replicate the present design with greater sample sizes. Finally, this study was conducted fully online and asynchronously, prioritizing participants’ variability over direct control. Because the participants completed the tasks at their own pace and responded to the survey online, it is possible that they paid less attention or dedicated less time to it than if it had been conducted in person. While fully online studies have been widely conducted in recent years, future studies should explore the possibility of response variation when an experimenter is present at the time of the study. 

## 5. Conclusions

The aim of the present study was to assess the predictive validity of individual differences in bilingual experience extracted via social network methodology to explain the sociolinguistic processes underlying theory of mind and metalinguistic awareness. The results herein suggest that some aspects of bilingual engagement at different points in the lifespan can affect the performance of perspective-taking ToM. Together, the results support the utility of an individual differences approach, specifically a multi-level approach as social network analysis, in future research examining the cognitive outcomes of bilingual experience. 

## Figures and Tables

**Table 1:** Sample demographic details (*N* = 89).

	Mean	SD	Frequency
**Age**	25.27	6.96	
**Female**			85%
**Male**			14%
**Third gender/non-binary**			1%
**Some college/high school diploma**			43%
**Associate Degree**			51%
**Bachelor’s Degree**			6%
**AoA L1**	1.26	1.35	
**AoA L2**	4.56	4.21	
**Speaks only L1**			6.5%
**Speaks L2**			85.7%
**Speaks > L2**			7.8%
**Years living in L1 country**	19.49	9.63	
**Years living in L2 country**	18.9	10.69	
**Single Culture**			49%
**Multicultural**			51%

*Note*: L1 = reported first language. L2 = reported second language. L3 = additional reported languages. AoA = age of acquisition.

**Table 2:** Descriptive statistics (*N* = 89).

Variables	Mean	SD	Range	Skew	Kurtosis
Metalinguistic Awareness	0.68	0.23	0–1.31	−0.13	0.62
ToM (MAD Curvature)	5.13	0.71	2.20–6.43	−1.61	3.03
Average L1 Use	44.89	24.39	0–100	−0.04	−1.32
Average L2 Use	33.72	17.32	0–100	−0.24	−1.12
Languages Across Lifespan	1.18	0.37	0–2.29	0.66	1.19
Ego Switching	2.46	0.72	0–4.18	0.03	0.42
Alters Switching	1.42	0.34	0–2	0.28	−1.22
Alters’ Languages during Childhood	0.69	0.36	0–2	0.00	−0.94
Alters’ Current Languages	0.91	0.31	0–2	−0.66	0.36
Total Languages	2.05	0.51	1–4	1.87	7.04
L1 Fluency	0.10	0.02	0–0.1	−2.28	7.75
L2 Fluency	0.09	0.02	0–0.1	−1.75	3.50
Alter Ties Languages	0.01	0.00	0–1	−0.62	−0.24
Alter Ties Languages during Childhood	0.01	0.00	0–1	0.06	−0.99
Alter Ties Switching	0.01	0.01	0–1	−1.15	0.51

**Table 3:** Bivariate correlations among main measures.

	1. ToM	2. L2 Use	3. Ego Switch	4. Alter Ties Language Childhood	5. Alter Ties Switch	6. Languages Lifespan	7. L2 F	8. MA
**1**	-							
**2**	**0.37**	-						
**3**	**0.29**	**0.37**	-					
**4**	**0.16**	0.01	−0.04	-				
**5**	**0.17**	0.09	**0.14**	**0.24**	-			
**6**	**0.13**	0.06	**0.16**	**0.33**	**0.27**	-		
**7**	**0.28**	0.77	**0.49**	0.00	0.08	−0.05	-	
**8**	−0.11	**−0.27**	**−0.14**	0.04	−0.02	**−0.21**	**−0.17**	-

*Note*: L2 F = second language fluency. MA = metalinguistic awareness. Bolded numbers indicate *p* ≤ 0.05.

**Table 4:** Multiple regression analysis of ToM with social network variables as predictors (*N* = 89).

	B	SE	*β*
**Mean L2 Use**	0.27	0.07	3.89 ***
**Ego Switching**	0.21	0.07	3.01 **
**Alter Ties Languages Childhood**	29.40	13.11	2.24 *
**Alter Ties Languages Switching**	11.95	8.76	1.37
**Languages Lifespan**	−0.02	0.13	−0.13
**L2 Overall Fluency**	−4.13	3.17	−1.31
**Metalinguistic Awareness**	−0.02	0.20	−0.12
**Adjusted R^2^**		0.18	

*Note*. *** ≤0.001, ** ≤0.01, * ≤0.05.

## Data Availability

Scripts and data will be made available on: https://osf.io/azmpk/ (accessed on 6 April 2022).
